# Neonate Human Remains: A Window of Opportunity to the Molecular Study of Ancient Syphilis

**DOI:** 10.1371/journal.pone.0036371

**Published:** 2012-05-02

**Authors:** Rafael Montiel, Eduvigis Solórzano, Nancy Díaz, Brenda A. Álvarez-Sandoval, Mercedes González-Ruiz, Mari Pau Cañadas, Nelson Simões, Albert Isidro, Assumpció Malgosa

**Affiliations:** 1 Laboratorio Nacional de Genómica para la Biodiversidad, Centro de Investigación y de Estudios Avanzados del Instituto Politécnico Nacional (CINVESTAV-IPN), Irapuato, Guanajuato, Mexico; 2 Unitat d'Antropologia, Departament de Biologia Animal, Biologia Vegetal i Ecologia, Universitat Autònoma de Barcelona, Bellaterra, Spain; 3 Departament de Biologia Molecular, General Lab, Barcelona, Spain; 4 Research Center for Natural Resources (CIRN), Department of Biology, University of the Azores, Ponta Delgada, Azores, Portugal; 5 Servei de Cirurgia Ortopèdica i Traumatologia (COT), Hospital Universitari del Sagrat Cor, Barcelona, Spain; University of Florence, Italy

## Abstract

Ancient DNA (aDNA) analysis can be a useful tool in bacterial disease diagnosis in human remains. However, while the recovery of *Mycobacterium* spp. has been widely successful, several authors report unsuccessful results regarding ancient treponemal DNA, casting doubts on the usefulness of this technique for the diagnosis of ancient syphilis. Here, we present results from an analysis of four newborn specimens recovered from the crypt of “La Ermita de la Soledad” (XVI–XVII centuries), located in the province of Huelva in the southwest of Spain. We extracted and analyzed aDNA in three independent laboratories, following specific procedures generally practiced in the aDNA field, including cloning of the amplified DNA fragments and sequencing of several clones. This is the most ancient case, reported to date, from which detection of DNA from *T. pallidum* subspecies *pallidum* has been successful in more than one individual, and we put forward a hypothesis to explain this result, taking into account the course of the disease in neonate individuals.

## Introduction

Syphilis was the most important chronic infectious disease that spread in Europe in the late 15th and early 16th centuries. Historical chroniclers relate that in March 1493 Columbus returned in triumph to the royal court in Barcelona, and while a second voyage was being prepared, a strange affliction broke out in the city. Everything indicated that, with the discovery of America in 1492, Columbus's sailors were infected there, and brought the disease back into Europe [1]. However, according to different scholars, syphilis appeared for the first time in Europe in 1493, in March in Rome, or in February in Naples [1]. Then, the period of time between the discovery of America and the outbreak of the epidemic, seems to be non tenable for developing the epidemic. In spite of the many publications on its origin, this issue is far from being resolved. While some authors claim a lack of evidence for syphilis in the Old World before the voyages of Columbus [2], a minority contend that syphilis occurred in Europe before the Christian Era [3], and some others find ancient bone remains that prove its ancient presence in Europe [4]–[8]. However, morphology-based diagnosis of treponemal disease in skeletal remains is not always reliable, and differentiating between different treponematoses, or even other entities such as leprosy, is very difficult. Ancient DNA (aDNA) analysis can be a useful tool in bacterial disease diagnosis in human remains [9] and, in the case of syphilis it can confirm the presence in the bones of DNA from *Treponema pallidum* subspecies *pallidum*, the causative agent of venereal syphilis [10]; helping to establish a reliable diagnosis. Nevertheless, while the recovery of DNA from *Mycobacterium* spp. has been widely successful [11]–[14], several authors report unsuccessful results regarding ancient treponemal DNA [15]–[17], casting doubts on the usefulness of this technique for the diagnosis of ancient syphilis. A hypothesis for this failure is based on the observation that in advanced stages of the disease, when bone marks are produced, Treponemal organisms “will most likely not be located in bone and the possibility of isolating them by PCR will be negligible” [17].

In general, syphilis is challenging to study in part because it cannot be cultured or genetically manipulated [18], and because the *T. pallidum* subspecies are morphologically indistinguishable by immunofluorescence or electron microscopy inducing similar histopathological changes and cross-reactive antibodies [19]. This makes diagnostic extremely difficult and therefore complicate further epidemiologic or phylogenetic analyses. However, molecular typing has proven to be useful in the specific detection of some of these subspecies [19]–[21]. In this sense, paleopathology could also benefit from molecular techniques for identification of ancient strains specific to syphilis and then generate information useful in the phylogenetic reconstruction of modern strains. This could also help to shed light on the historical development of the disease, regarding continental origin, geographic distribution and epidemiology.

Here, we present results from an analysis of newborn specimens sharing similar osteoporotic pathology. They were recovered from the crypt of “La Ermita de la Soledad”, located in the province of Huelva in the southwest of Spain, the province from where the first Columbus expedition departed to America in 1492. This Hermitage was built between the XVI and XVII centuries [22]. Although the exact date of its construction is unknown, the human remains buried in the crypt cannot be dated before the discovery of the Americas. Nevertheless, this is the most ancient case, reported to date, from which detection of Treponemal DNA has been successful. We put forward a hypothesis on why we succeeded taking into account the course of the disease in neonate individuals.

## Results

### Individual's Genetic Characterization

**Figure 1:**
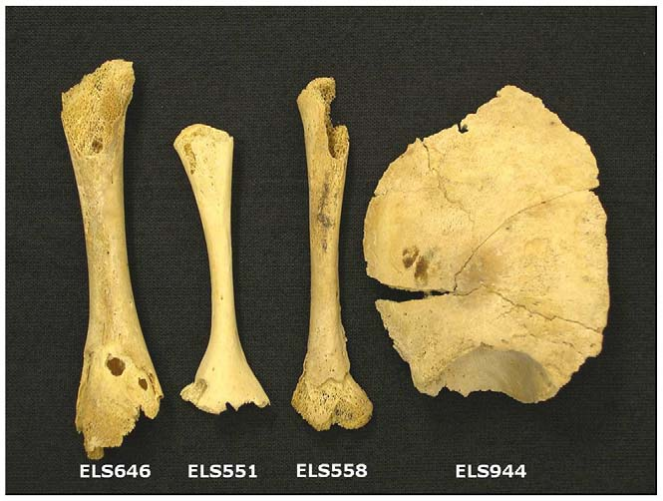
The presence of two left humeri (ELS 551 and ELS 558) indicates that at least two individuals are represented in these remains. Molecular data confirmed the presence of at least two individuals, one that might be represented by ELS 551, ELS646, and ELS 944, and one represented by ELS558 (see results).

Four newborn specimens with similar osteoporotic pathology were analyzed: one left femur (ELS 646), two left humeri (ELS 551 and ELS 558) and one right hemi-frontal (ELS 944) (Fig. 1). We obtained partial sequences of the mitochondrial Hypervariable Segment I (HVSI) for each of the four specimens analyzed (Table 1). HVSI is the most informative segment of the control region of mitochondrial DNA, and the most used in human population studies. For all but one of the specimens (ELS646) independent replication was accomplished in at least two laboratories. From the pattern of substitutions observed among the sequences, we can identify at least two different sequences. Specimens ELS 551, ELS646, and ELS 994 share the 16270T, 16311C distinctive motif, indicating they belong either to the mitochondrial haplogroup U5 or to haplogroup K2; while ELS 558 is differentiated by the absence of this motif having a haplotype identical to the Cambridge Reference Sequence (CRS) for this fragment.

**Table 1:** Partial mitochondrial DNA sequences (16210–16319) obtained from the analyzed specimens in the different laboratories involved in the study.

Lab Sample	UAB	UAÇ	LANGEBIO
ELS551	nd	16270T, 16311C	16270T, 16311C
ELS558	CRS	CRS	nd
ELS646	16270T, 16311C	na	na
ELS944	16270T, 16311C	na	16270T, 16311C

Only differences to the CRS are shown. ND, not determined because amplification was not possible in the independent extract obtained at the specified laboratory; NA, amplification was not attempted (sample was not sent) at the specified laboratory; CRS, identical to the Cambridge Reference Sequence.

In summary, along with morphological information, HVSI sequences indicate these samples come from at least two different individuals, one that might be represented by ELS 551, ELS646, and ELS 944, and another represented by ELS 558.

### *Treponema pallidum* DNA amplification

At the UAB laboratory, the four samples (ELS 551, ELS 558, ELS 646 and ELS 944) amplified for an *arp* gene fragment (see Material and Methods). For all samples, the 106 bp fragment was digested with *Nae*I, after which, the expected fragment of 84 bp was observed, while the 22 bp fragment was not, due to the presence of primer-dimer products (data submitted but not shown). As most of the amplification product was used in the digestion assay, the sequencing of this fragment was not attempted, considering also that the identity of the fragment was confirmed by the presence of the *Nae*I restriction site at the expected position.

**Figure 2:**
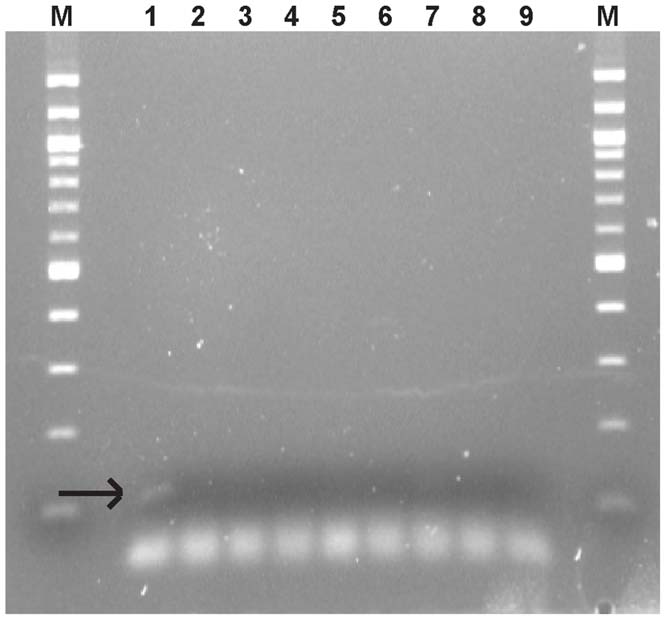
Amplification of the *arp* gene fragment from the extracts obtained at the UAÇ. M, molecular weight marker, 100 bp DNA ladder (Sigma); 1, ELS 551; 4,7, ELS 558; 2,5 unaffected ancient samples; 3,6,8 extraction blanks (KE); 9, PCR blank (K−).

**Figure 3:**
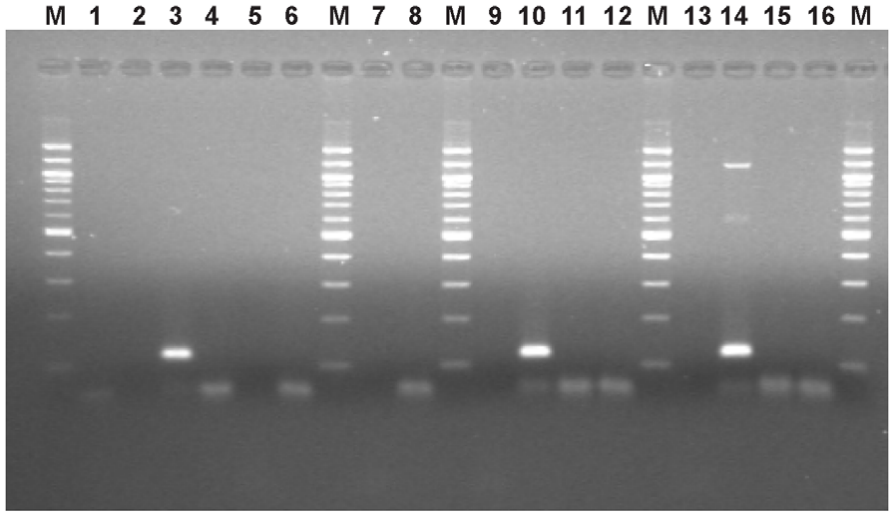
Amplification of the 119-bp fragment of the 5′UTR of the 15-kDa lipoprotein gene of *T. pallidum*, from DNA extracts obtained at the UAÇ. M, molecular weight marker, 100 bp DNA ladder (Sigma); 3, ELS 551; 10,14, ELS 558; 1,2,4,5,7,9,11,13 unaffected ancient samples; 6,8,12,15 extraction blanks (KE); 16, PCR blank.

At the UAÇ, DNA from specimens ELS 551 and ELS 558 was independently extracted and analyzed. A weak amplification of the *arp* gene fragment was obtained only for ELS 551 (Fig. 2). The band was cloned and 3 positive clones were sequenced in both directions. The sequence from the three inserts was identical among them, and to the sequence of *T. pallidum* spp. (Figure S1). The restriction site for *Nae*I was identified at the expected position. We have not a definite explanation regarding why ELS 558 was not amplified for this fragment at the UAÇ, considering it worked at the UAB. There are many reasons that could explain this result, including differences in PCR equipments or primers qualities, and/or differences in the content of inhibitors in the extracts used, since the attempts were made from independent extracts at each laboratory. From the amplification of the 5′UTR fragment of the 15-kDa lipoprotein gene, clear bands were obtained from both specimens ELS 551 and ELS 558, and cloned (Fig. 3). From ELS551 three clones were sequenced in both directions. From ELS558 three clones were also sequenced in both strands. The 119 bp sequences from all six clones were identical to the sequence of *T. pallidum* subsp. *pallidum* (Figure S2). Therefore, the *Eco*47III restriction site characteristic of this subspecies was present in all the sequences.

At Langebio, amplification of the 5′UTR fragment of the 15-kDa lipoprotein gene was obtained from the three analyzed specimens (ELS 551, ELS 558 and ELS 944). From ELS 551 we obtained sequences from 14 clones, from ELS 558 six clones were sequenced, and from ELS 944 only one clone was sequenced. All sequences were identical to the sequence of *T. pallidum* subsp. *pallidum* (Figure S3).

## Discussion

In this study we amplified ancient Treponemal DNA from four specimens belonging to at least 2 different individuals. This represents the highest rate of success in amplifying ancient Treponemal DNA. Previously, just one ancient individual yielded positive result [10], despite several attempts in dozens of individuals analyzed [15]–[17]. The main difference between our study and previous reports lies in the fact that we analyzed neonate remains, with clear signs of being affected by congenital syphilis (CS). This lead us to hypothesize that in remains from CS-affected neonates the probability of preservation of Treponemal DNA is highly increased, due to a rapid dissemination in the skeleton of a high number of spirochetes, which after the death would leave their DNA that would be preserved by its association to hydroxyapatite in bones.

Recently a newborn infant affected by CS showed long-bone lesions upon X-ray examination [23], and Armangil *et al*. [24] reported a newborn infant with unusual clinical findings of CS such as a non-fluctuant mass surrounding the left calf, without any additional system involvement such as hepatic or skin involvement or lymph nodes. Furthermore, a study analyzing 121 infants below 3 months of age, affected by CS, also found bone damage in 54.3% of the cases [25]. This indicates that newborns are sensitive to bone damage at early stages of the disease, supporting the findings of Schneider (cited by [26]) who reported that, in congenitally syphilitic infants, resting spirochetes may be found in the bone cell lacunae, and demonstrate the rapid spread of *T. pallidum* throughout the whole skeleton in CS. In 1931 Bauer demonstrated the presence of *T. pallidum* in the pulp, the dentinoid, the dentinal tubules, the tooth sac, the ameloblast layer and even in the stellate reticulum, by microscopic studies in four congenitally syphilitic fetuses and infants [27]. Further studies on the jaws of six congenitally syphilitic but nonmacerated subjects (four fetuses of 6, 8, and 9 months, and two infants of 2 1/2 weeks and 1 1/2 months of age) demonstrated the presence of *T. pallidum* in the bone and tooth tissues, although it was noted that the older the infant the fewer were the spirochetes observed [27]. Some years later Je *et al*. [28] found *T. pallidum* spirochetes in the ameloblast layer, stellate reticulum of enamel epithelium, odontoblast layer, and predentin layer, analyzing two cases of CS.

These data support the hypothesis that in neonates the number of spirochetes in the skeleton is good enough to warrant their DNA preservation, and that the younger the individual affected by CS, the bigger the probability of amplifiable DNA preservation. This is in contrast to the case of adults affected by venereal syphilis, in which the number of spirochetes in bones is reduced as the disease advance to later stages. Furthermore, von Hunnius *et al*. [17] infected rabbits with *T. pallidum pallidum* in order to assess the feasibility of isolating treponemal DNA from bones. Their results showed that although treponemal DNA could be isolated from the site of injection in the “chronic rabbit”, it could only be isolated from the bone during the acute stage (not in advanced stages of the disease) [17].

It is worth noting that a number of studies have shown the presence of bone lesions in early syphilis [29]–[31], and this opens up the possibility that affected skeletons of young adults, who died during the early stages of syphilis, might also contain amplifiable *T. pallidum* DNA. However, the doubt remains on how to identify those cases before attempting destructive analysis. Our study demonstrates that neonates affected by CS are in fact a good source of Treponemal ancient DNA.

## Material and Methods

### Samples

The remains of at least eighty individuals of both sexes were recovered during archaeological excavation works prior to restoring “La Ermita de la Soledad” crypt. Seventeen of these individuals were neonates ranging in age from birth to six months at the time of death [32]. Historical data indicates that the crypt was built between the XVI and XVII centuries [22]. The analysis of this material was originally requested by Diputación de Huelva (Autonomous Government of Huelva Province, Spain), therefore no specific permits were required for the studies described below.

In this study, four newborn specimens with similar osteoporotic pathology were analyzed: one left femur (ELS 646), two left humeri (ELS 551 and ELS 558) and one right hemi-frontal (ELS 944). Age at death was diagnosed from bone length [33]–[35] along with the thickness and dimensions of cranial vault bones [36], [37]. The specimens were studied both morphologically and radiologically. A high degree of osteoporoses, detachment of the outer layer of the cortical bone, osteolytic juxtametaphysial areas and distal epiphysis destruction were observed. Integrating this data, differential diagnosis was conducted taking into account the subject's age at death and the probability of suffering the illness considering the site's geographical location and chronology; taken together, these observations supported the hypothesis of an infectious etiology such as congenital treponematosis or haematogenous osteomyelitis [38].

Initial molecular analysis was conducted at the Universitat Autònoma de Barcelona (UAB). Subsequently, bone fragments from two left humeri (ELS 551 and ELS 558) were sent to the Universidade dos Açores (UAÇ, Portugal), for independent replication. Samples from the same left humeri and a fragment from the right hemi-frontal (ELS944) were also sent to the Laboratorio Nacional de Genómica para la Biodiversidad (Langebio, CINVESTAV-IPN, Mexico).

### DNA Extraction and Purification

In the three laboratories involved in the present study, DNA was extracted according to the methodology developed at the aDNA laboratory of the Biological Anthropology Unit of the UAB, with the conditions of sterility and the suitable precautionary measures previously described [39]. The extraction process started with the elimination of the outer bone surface, followed by bone powdering by using a micro motor with a hand piece and diamond drill for dentist use; washings with EDTA; incubation at 37°C with extraction buffer and Proteinase K; DNA extraction with phenol/chloroform; and centrifugation dialysis with sterile water, and concentration with a Centricon 30 filter Millipore®. Finally, the extracts were stored at 4°C for four days to overcome PCR inhibitors [40], [41]. In order to monitor contamination, extraction blanks (KE), containing only reagents, were subjected to analysis. PCR negative controls (K−) were also included.

At the UAB and Langebio, sample preparation, DNA extraction and PCR set-up were performed in laboratories dedicated specifically to ancient DNA, positively pressurized and physically isolated from the PCR thermal cycler and the laboratories for post-PCR or any other molecular biology research; additionally, full-body suits, breathing masks and protective lenses were used. At the UAÇ, DNA extraction was conducted in a biological security hood, where no previous DNA extraction of any organisms had been performed before. PCR reactions were prepared in a different cabinet (horizontal filtered laminar flux), located in a different laboratory and dedicated solely to this purpose.

In all three laboratories, no work with modern *Treponema* spp. had been previously conducted.

### Individual's Genetic Characterization

According to anatomical information, the four bone fragments analyzed belonged to at least two individuals (ELS 551 and ELS 558 are two left humeri). To clarify if the other remains represented different individuals, the hypervariable segment I (HVSI) of the mitochondrial control region was amplified. At the UAB the primers L15997-H16401, or two sets of overlapping primers (L15997-H16254 and L16209-H16401) were used. Amplified fragments were purified and directly sequenced in both strands. At the UAÇ and at Langebio, primers L16190-H16209 and L16320-H16339 were used. Amplified fragments were cloned and sequenced in both strands. Only the region comprising 16210–16319 was obtained in at least two independent laboratories and this is the region presented here. Numbers refers to nucleotide positions in the Cambridge Reference Sequence (CRS) [42].

### *Treponema pallidum* DNA amplification

At the UAB, *T. pallidum* DNA was amplified with specific primers designed for this study (F284 5′-GTGATCCTCTGTCATCCCCG-3′ and R370 5′-TCCACCTCACGAGACAAAGG-3′), that amplify a fragment of 106 bp, between positions 284 and 370, of the acidic repeat protein gene (*arp*) [43]. A Blast search [44] performed at the NCBI GenBank showed that this fragment presents 100% of similarity only with *T. pallidum* subspecies, with an E-value of 2×10^−47^ for all of the subspecies of *T. pallidum* that were matched. The primers were tested independently at General lab facilities (Barcelona) by using DNA from *T. pallidum* subsp. *pallidum* Nichols strain as a positive control. Although both Blast and experimental results gave us confidence on the specificity of this fragment to *T. pallidum* subspecies, it is not useful to discriminate among subspecies, and therefore a new set of primers was also used in subsequent experiments (see below). Amplification of the *arp* fragment was performed in a 50 µl volume. Thermal cycler conditions consisted of an initial denaturation at 94°C for 5 min, followed by 39 cycles at 94°C for 50 seconds, 55°C for 1 min and 72°C for 1 min and final extension at 72°C for 5 min. Blank controls (K−) were included in all PCR assays. The target fragment presents a *Nae*I restriction site that produces two fragments of 84 and 22 bp after digestion. Digestions with *Nae*I were performed according to the supplier instructions (New England Biolabs).

At the UAÇ, besides the fragment of the *arp* gene, amplification of a 119-bp fragment of the 5′ untranslated region (UTR) of the 15-kDa lipoprotein gene that contains an *Eco*47III restriction site specific to *T. pallidum* subsp. *pallidum* [20] was also attempted; using the primers and amplification conditions described by Kolman *et al*. [10]. The specificity of the *Eco47*III restriction site to the *pallidum* subspecies was previously demonstrated by Centurion-Lara and coworkers [20]. We further performed a Blast search and found that 100% of similarity, including the recognition sequence for *Eco*47III (AGCGCT), was found only for *T. pallidum* subspecies *pallidum*, with an E-value of 1×10^−54^. Other *T. pallidum* subspecies or other species of *Treponema*, failed to present the *Eco47*III restriction site. PCR products from both the *arp* gene and the 5′UTR of the 15-kDa lipoprotein gene were cloned into TOPO-TA (Invitrogen). Positive clones were sequenced in both strands using M13 forward and reverse primers.

Due to its specificity to *T. pallidum* subspecies *pallidum*, in Langebio only the amplification of the 119-bp fragment of the 15-kDa lipoprotein gene was conducted. PCR products were cloned into pJET1.2/blunt (Fermentas) and positive clones were sequenced in both strands using pJET1.2 forward and reverse sequencing primers.

## Supporting Information

Figure S1 **Acidic Repeat Protein (** ***arp*** **) gene fragment of** ***Treponema pallidum*** **subspecies** ***pallidum*** **, aligned with clone sequences from sample ELS551, obtained at the Universidade dos Açores (Portugal).** A box shows the *Nae*I restriction site used to analyze amplifications at the Universitat Autònoma de Barcelona (Spain).
(JPG)

Figure S2 **5′UTR fragment of the 15-kDa lipoprotein gene of** ***Treponema pallidum*** **subspecies** ***pallidum*** **, aligned with clone sequences from samples ELS551 and ELS558, obtained at the University of the Azores (Portugal).** A box shows the *Eco*47III restriction site, specific to this subspecies.
(JPG)

Figure S3 **5′UTR fragment of the 15-kDa lipoprotein gene of** ***Treponema pallidum*** **subspecies** ***pallidum*** **, aligned with clone sequences from samples ELS551, ELS558, and ELS944, obtained at Langebio, CINVESTAV-IPN (Mexico).** A box shows the *Eco*47III restriction site, specific to this subspecies.
(JPG)
