# Supplementary figures and images for: Neonate Human Remains: A Window of Opportunity to the Molecular Study of Ancient Syphilis
Source: PLoS One. 2012 May 2;7(5):e36371. doi: 10.1371/journal.pone.0036371 (PMC3342265; doi:10.1371/journal.pone.0036371)

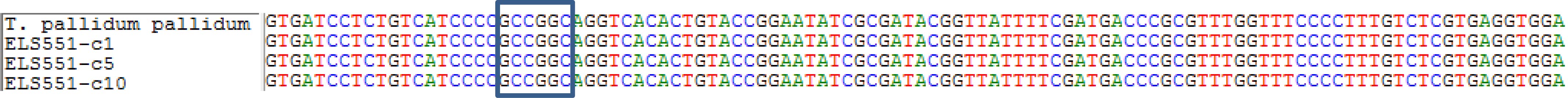

Supplement: Figure S1 — Acidic Repeat Protein ( arp ) gene fragment of Treponema pallidum subspecies pallidum , aligned with clone sequences from sample ELS551, obtained at the Universidade dos Açores (Portugal). A box shows the NaeI restriction site used to analyze amplifications at the Universitat Autònoma de Barcelona (Spain). (JPG) [file pone.0036371.s001.jpg]

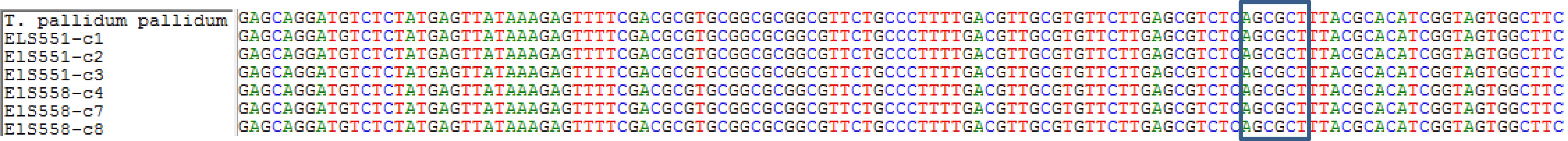

Supplement: Figure S2 — 5′UTR fragment of the 15-kDa lipoprotein gene of Treponema pallidum subspecies pallidum , aligned with clone sequences from samples ELS551 and ELS558, obtained at the University of the Azores (Portugal). A box shows the Eco47III restriction site, specific to this subspecies. (JPG) [file pone.0036371.s002.jpg]

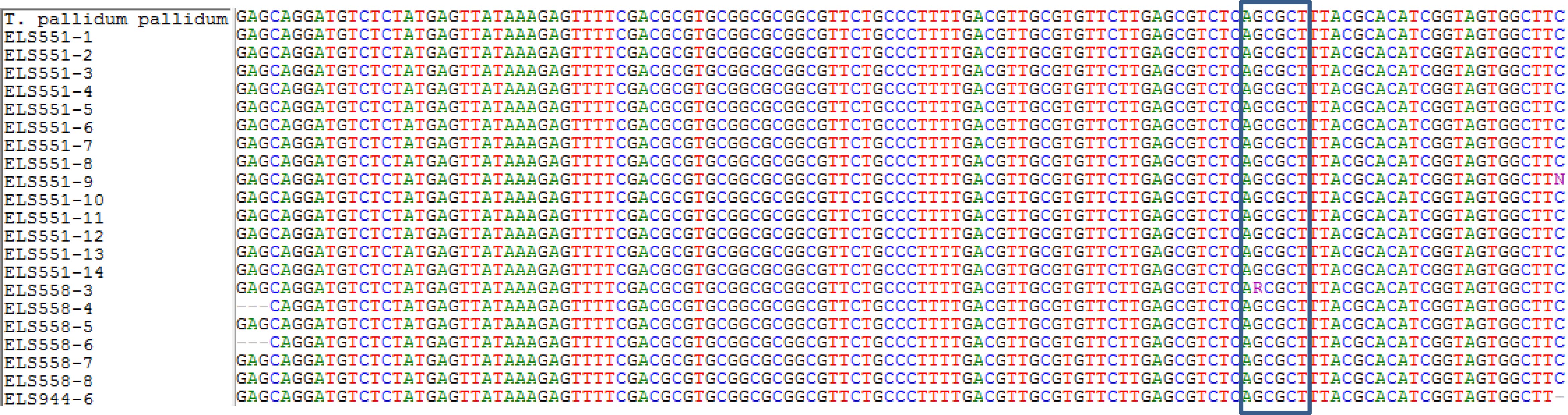

Supplement: Figure S3 — 5′UTR fragment of the 15-kDa lipoprotein gene of Treponema pallidum subspecies pallidum , aligned with clone sequences from samples ELS551, ELS558, and ELS944, obtained at Langebio, CINVESTAV-IPN (Mexico). A box shows the Eco47III restriction site, specific to this subspecies. (JPG) [file pone.0036371.s003.jpg]
